# Supplementary material for: RIG-I is an intracellular checkpoint that limits CD8+ T-cell antitumour immunity
Source: EMBO Mol Med. 2024 Sep 25;16(11):3005–25. doi: 10.1038/s44321-024-00136-9 (PMC11555380; doi:10.1038/s44321-024-00136-9)
Supplement: Supplementary file 1 — Appendix [file 44321_2024_136_MOESM1_ESM.pdf]

RIG-I is an intracellular checkpoint that limits $CD8^+$ T-cell antitumour immunity

Xiaobing Duan[1, 5*✉], Jiali Hu[1*], Yuncong Zhang[1*], Xiaoguang Zhao[1], Mingqi Yang[1], Taoping Sun[3], Siya Liu[4], Xin Chen[5], Juan Feng[5], Wenting Li[1], Ze Yang[1], Yitian Zhang[1], Xiaowen Lin[1], Dingjie Liu[1], Ya Meng[1], Guang Yang[1], Qiuping Lin[3], Guihai Zhang[6], Haihong Lei[7], Zhengsheng Yi[7], Yanyan Liu[1], Xiaobing Liang[8], Yujuan Wu[9], Wenqing Diao[9], Zesong Li[10], Haihai Liang[1, 11], Meixiao Zhan[1, 2], Hong-Wei Sun[1✉], Xian-Yang Li[1, 12✉], and Ligong Lu[1, 2✉]

Table of contents

Appendix Figure S1 2
Appendix Figure S2 3
Appendix Table S1 4

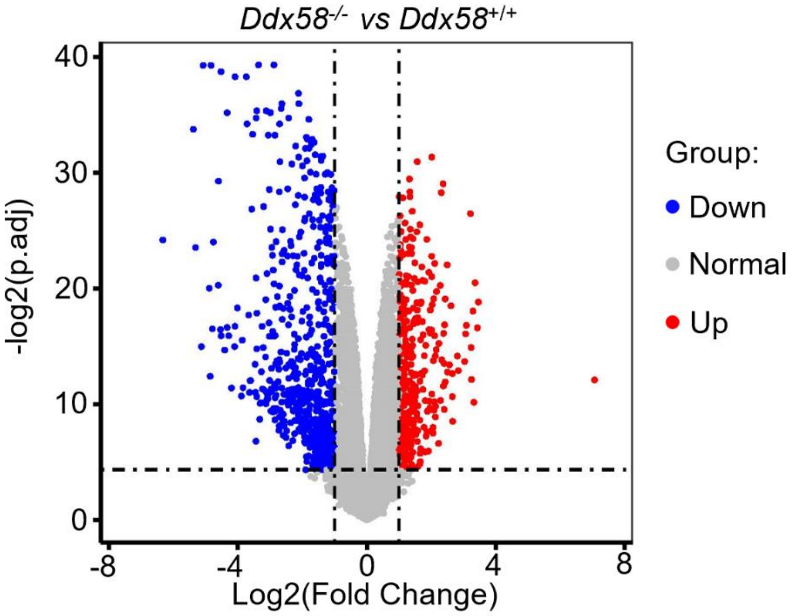

Appendix Figure S1. Volcano plot showing differentially expressed genes (DEGs) in *Ddx58$^{-/-}$* vs. *Ddx58$^{+/+}$* models. The upregulated genes (red) and the downregulated genes (blue) with a fold change ≥2 and with *P*.adjust < 0.05 are shown.

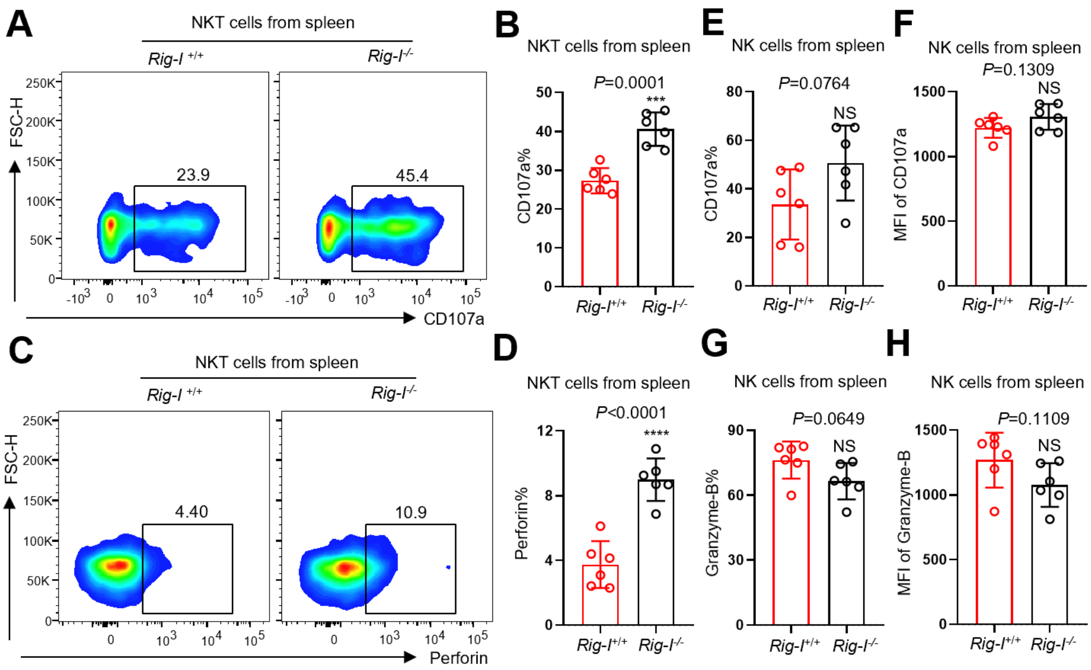

Appendix Figure S2. Knocking out RIG-I can enhance the functional antitumour capabilities of certain innate immune cells. (**A-D**) Flow cytometry analysis of CD107a and perforin levels in NKT cells from *Rig-I*$^{+/+}$/*Rig-I*$^{-/-}$ mouse spleens *ex vivo*. (**E-H**) Flow cytometry analysis of CD107a and granzyme-B levels in NK cells from *Rig-I*$^{+/+}$/*Rig-I*$^{-/-}$ mouse spleens *ex vivo*.

Data information: The data represented different numbers (n=6) of biological replicates, and were shown as the means ± SEMs. Two-tailed unpaired Student's t test was used in (**B, D, E, F and H**). A two-tailed Mann–Whitney U test was used in (**G**). ***P< 0.001, ****P< 0.0001, and NS, not significant compared with the *Rig-I*$^{+/+}$ group. Source data are available online for this figure.

Appendix Table S1. Primers used for the construction of molecular clones.

Primer	Sequence
sgRNA-RIG-I-1 (F)	CACCAAACAACAAGGGCCCAATGG
sgRNA-RIG-I-1 (R)	AAACCCATTGGGCCCTTGTTGTTT
sgRNA-RIG-I-2 (F)	CACCGGAACAAGTTCAGTGAACTG
sgRNA-RIG-I-2 (R)	AAACCAGTTCACTGAACTTGTTCC
sgRNA-RIG-I-3 (F)	CACCGATCAGAAATGATATCGGTT
sgRNA-RIG-I-3 (R)	AAACAACCGATATCATTTCTGATC
sgRNA-*Rig-I*-1 (F)	CACCCAGGCTGAGAAGAACAACAA
sgRNA-*Rig-I*-1 (R)	AAACTTGTTGTTCTTCTCAGCCTG
sgRNA-*Rig-I*-2 (F)	CACCCCAGTACCTGTTGAAGCTGC
sgRNA-*Rig-I*-2 (R)	AAACGCAGCTTCAACAGGTACTGG
sgRNA-*Rig-I*-3 (F)	CACCCAGTTGATCCAAATGATATC
sgRNA-*Rig-I*-3 (R)	AAACGATATCATTTGGATCAACTG
