# Supplementary figures and images for: RIG-I is an intracellular checkpoint that limits CD8+ T-cell antitumour immunity
Source: EMBO Mol Med. 2024 Sep 25;16(11):3005–25. doi: 10.1038/s44321-024-00136-9 (PMC11555380; doi:10.1038/s44321-024-00136-9)

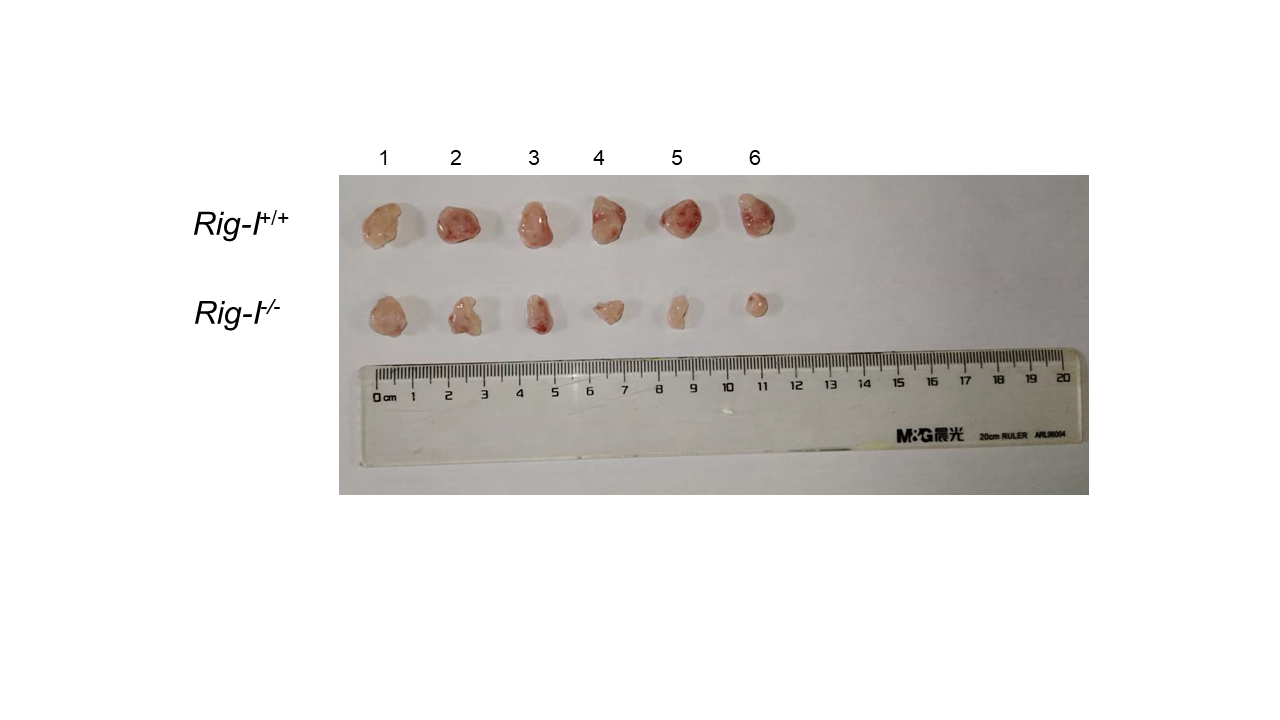

Supplement: Supplementary file 7 — Source data Fig. 4 [file 44321_2024_136_MOESM7_ESM.zip › Source Data Figure 4/4D/Figure 4D.tif]

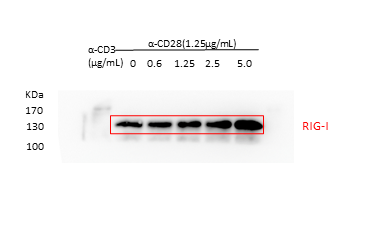

Supplement: Supplementary file 8 — Source data Fig. 5 [file 44321_2024_136_MOESM8_ESM.zip › Source Data Figure 5/5A/5A-RIG-I.tif]

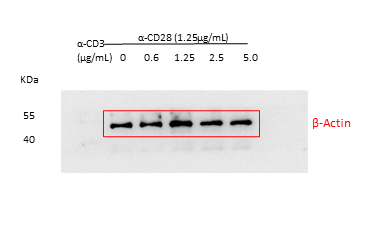

Supplement: Supplementary file 8 — Source data Fig. 5 [file 44321_2024_136_MOESM8_ESM.zip › Source Data Figure 5/5A/5A-a┬-Actin.tif]

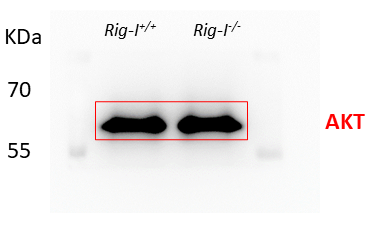

Supplement: Supplementary file 8 — Source data Fig. 5 [file 44321_2024_136_MOESM8_ESM.zip › Source Data Figure 5/5F/5F-AKT.tif]

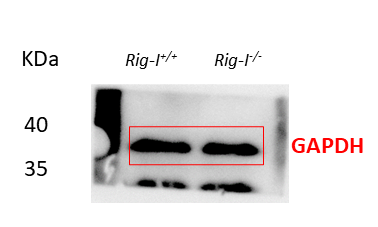

Supplement: Supplementary file 8 — Source data Fig. 5 [file 44321_2024_136_MOESM8_ESM.zip › Source Data Figure 5/5F/5F-GAPDH.tif]

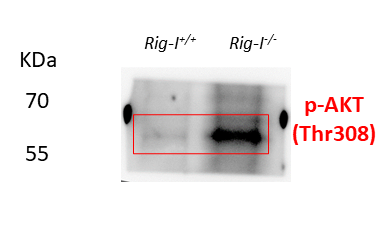

Supplement: Supplementary file 8 — Source data Fig. 5 [file 44321_2024_136_MOESM8_ESM.zip › Source Data Figure 5/5F/5F-p-AKT Thr308.tif]

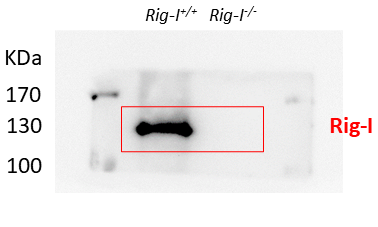

Supplement: Supplementary file 8 — Source data Fig. 5 [file 44321_2024_136_MOESM8_ESM.zip › Source Data Figure 5/5F/5F-RIG-I.tif]

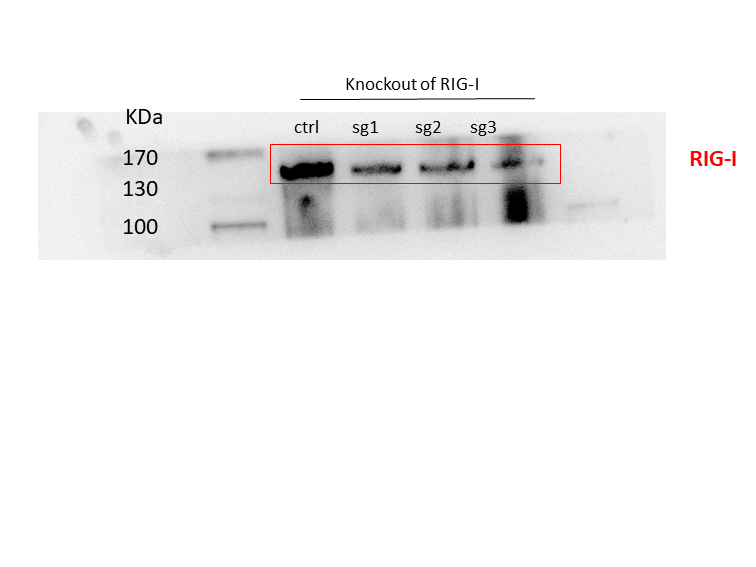

Supplement: Supplementary file 8 — Source data Fig. 5 [file 44321_2024_136_MOESM8_ESM.zip › Source Data Figure 5/5G/5G-RIG-I.tif]

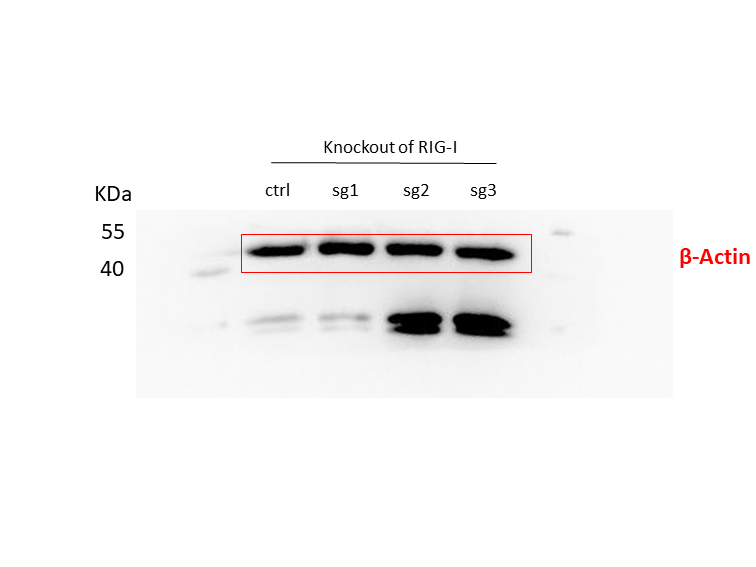

Supplement: Supplementary file 8 — Source data Fig. 5 [file 44321_2024_136_MOESM8_ESM.zip › Source Data Figure 5/5G/5G-a┬-Actin.tif]

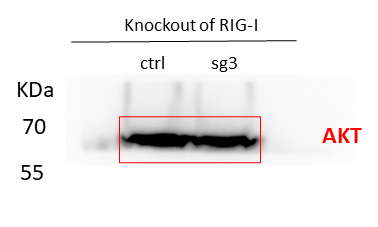

Supplement: Supplementary file 8 — Source data Fig. 5 [file 44321_2024_136_MOESM8_ESM.zip › Source Data Figure 5/5H/5H-AKT.tif]

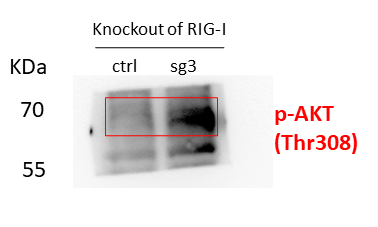

Supplement: Supplementary file 8 — Source data Fig. 5 [file 44321_2024_136_MOESM8_ESM.zip › Source Data Figure 5/5H/5H-p-AKT Thr308.tif]

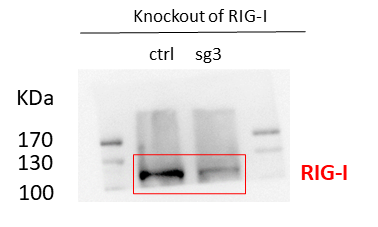

Supplement: Supplementary file 8 — Source data Fig. 5 [file 44321_2024_136_MOESM8_ESM.zip › Source Data Figure 5/5H/5H-RIG-I.tif]

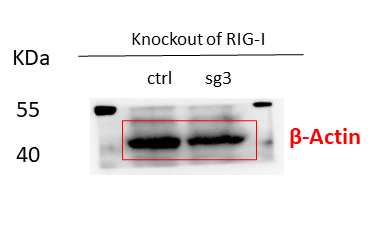

Supplement: Supplementary file 8 — Source data Fig. 5 [file 44321_2024_136_MOESM8_ESM.zip › Source Data Figure 5/5H/5H-a┬-Actin.tif]

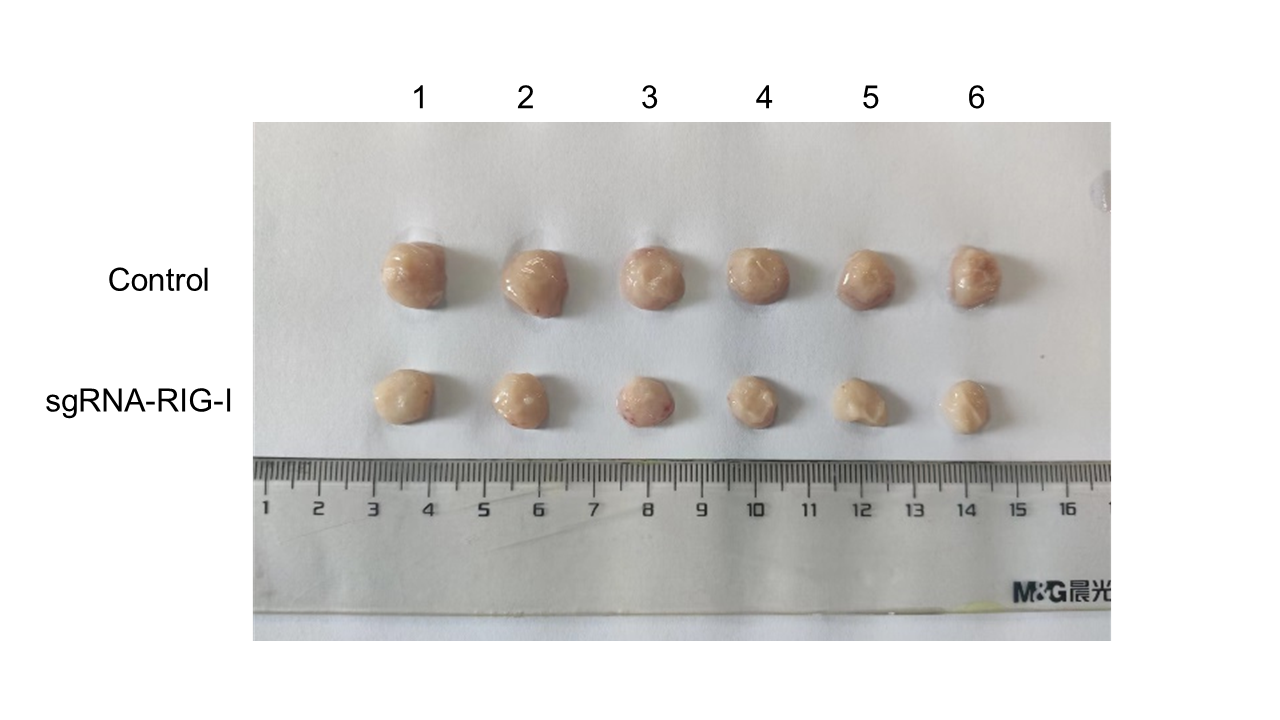

Supplement: Supplementary file 9 — Source data Fig. 6 [file 44321_2024_136_MOESM9_ESM.zip › Source Data Figure 6/6C/Figure 6C.tif]

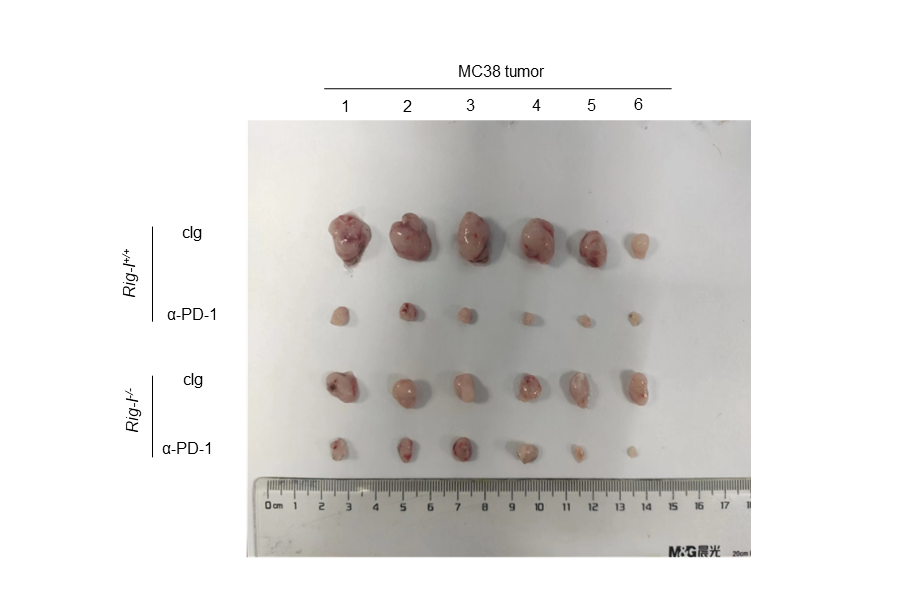

Supplement: Supplementary file 10 — Source data Fig. 7 [file 44321_2024_136_MOESM10_ESM.zip › Source Data Figure 7/7C/Figure 7C.tif]

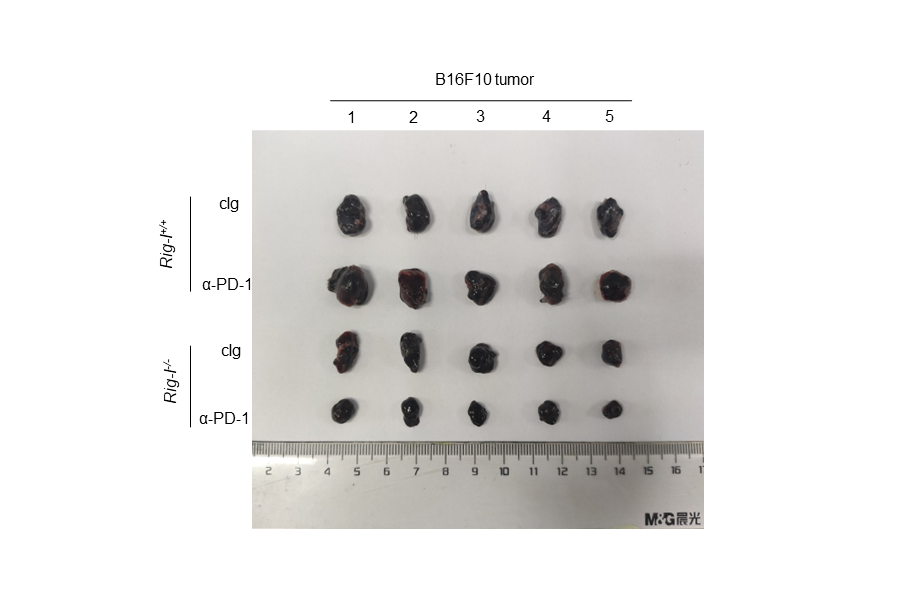

Supplement: Supplementary file 10 — Source data Fig. 7 [file 44321_2024_136_MOESM10_ESM.zip › Source Data Figure 7/7F/Figure 7F.tif]
