# Supplementary material for: RIG-I is an intracellular checkpoint that limits CD8+ T-cell antitumour immunity
Source: EMBO Mol Med. 2024 Sep 25;16(11):3005–25. doi: 10.1038/s44321-024-00136-9 (PMC11555380; doi:10.1038/s44321-024-00136-9)
Supplement: Supplementary file 13 — Expanded View Figures [file 44321_2024_136_MOESM13_ESM.pdf]

Expanded View Figures

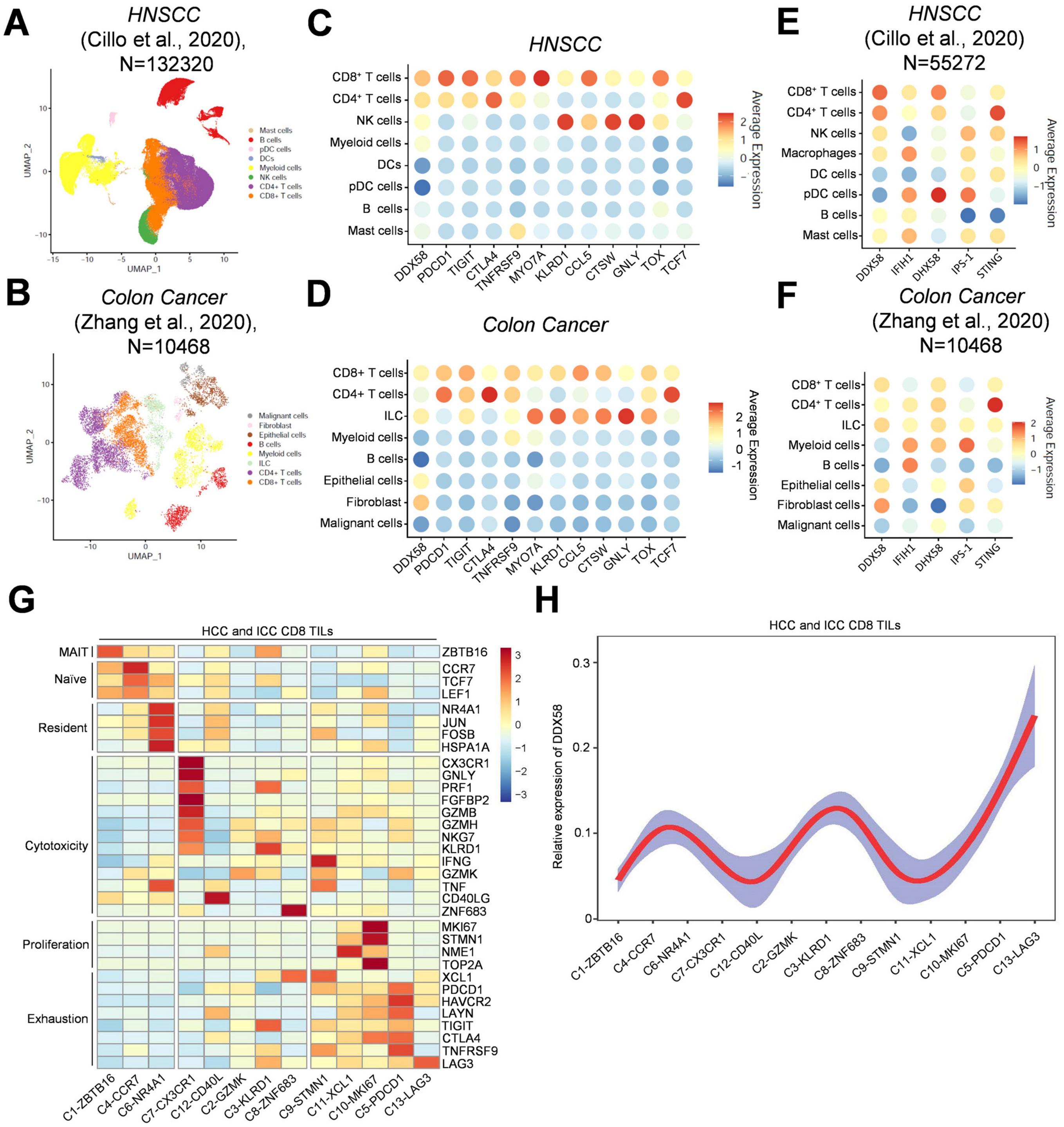

Figure EV1. Screening single-cell sequencing data and verification of RIG-I upregulation in CD8⁺ T cells infiltrating the TME.

(**A, B**) Cluster analysis of cell populations in HNSCC and colon cancer, and expression levels of the *DDX58* gene in each subpopulation. (**C, D**) Relative expression levels of *DDX58*, *PDCD1*, *TIGIT*, *CTLA-4* and *TOX*-associated exhaustion genes in each subpopulation in HNSCC (**C**) and colon cancer (**D**). (**E, F**) Relative expression levels of *DDX58*, *IFIH1*, *DHX58*, *IPS-1* and *STING* in HNSCC (**E**) and colon cancer (**F**) tissues. (**G**) Heatmap indicating the expression of selected gene sets in CD8⁺ T-cell subtypes infiltrating HCC and ICC. (**H**) Line chart showing the relative expression patterns of DDX58 in each CD8⁺ T-cell subtype. Source data are available online for this figure.

© The Author(s)

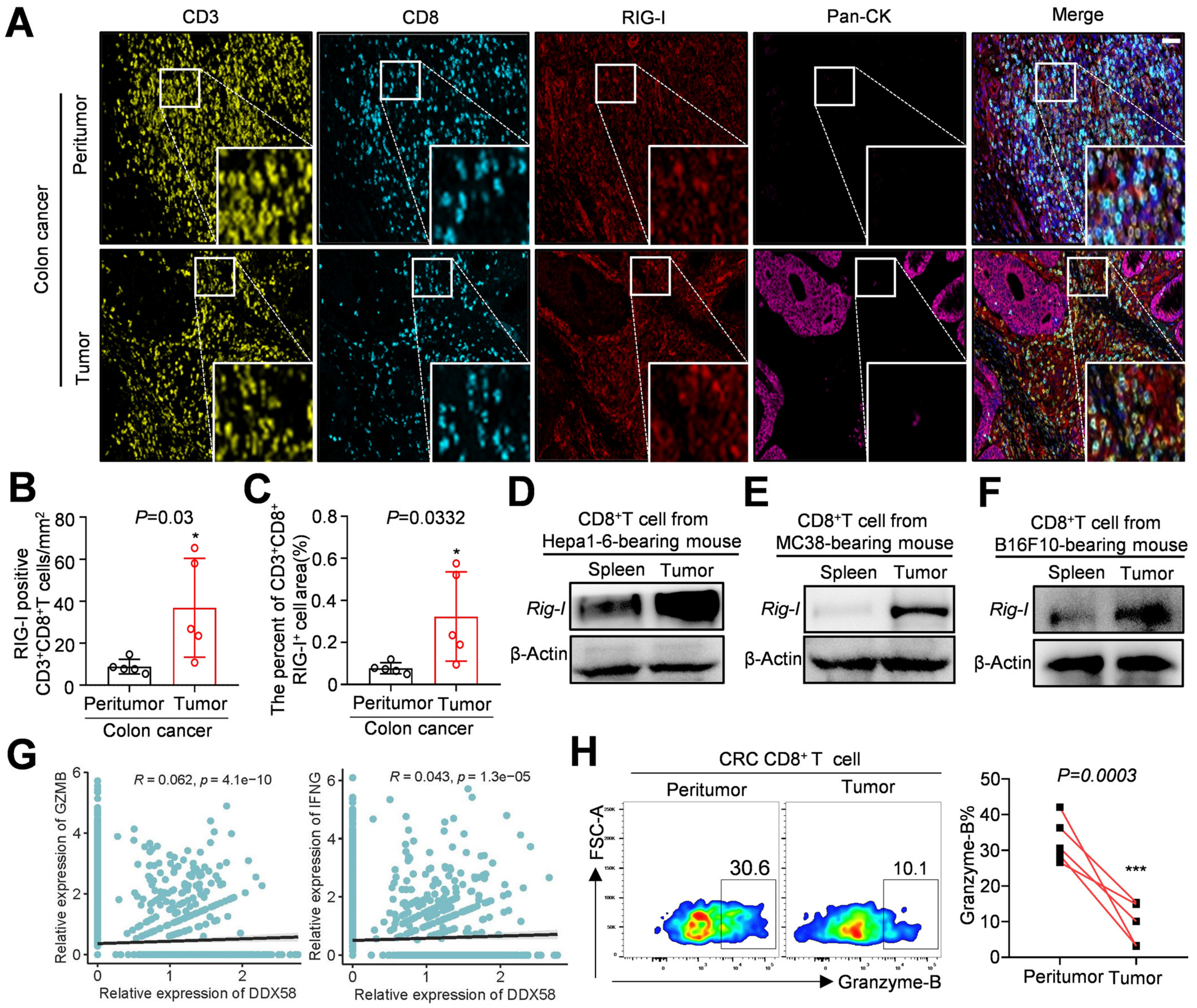

Figure EV2. The upregulation of RIG-I expression was validated in CD8$^+$ T cells infiltrating the TME.

(**A–C**) Multiple immunohistochemistry and statistical analysis of human colon cancer specimens. White scale bar = 50 μm. (**D–F**) The expression of *Rig-I* in CD8$^+$ T cells from the spleens and tumours of different tumour-bearing mice was detected using Western blotting. (**G**) Correlation analysis of DDX58 expression with GZMB and IFNG expression. (**H**) The expression of granzyme-B secreted by infiltrating CD8$^+$ T cells in the peritumor and tumour tissues of CRC by flow cytometry after stimulating with PMA/ionomycin and GolgiStop. Data information: The data represented different numbers ($n = 5$) of biological replicates and were shown as the means ± SEMs. Two-tailed unpaired Student's test was used in (**B**) ($P = 0.03$), (**C**) ($P = 0.0332$). The Pearson correlation test was used in (**G**) (Exact *p* values were reported on graphs). The values of *P* and *R* are shown in the figure. Two-tailed paired Student's test was used in (**H**) ($P = 0.0003$). *$P < 0.05$, ***$P < 0.001$, compared with the peritumoral group. Source data are available online for this figure.

© The Author(s)

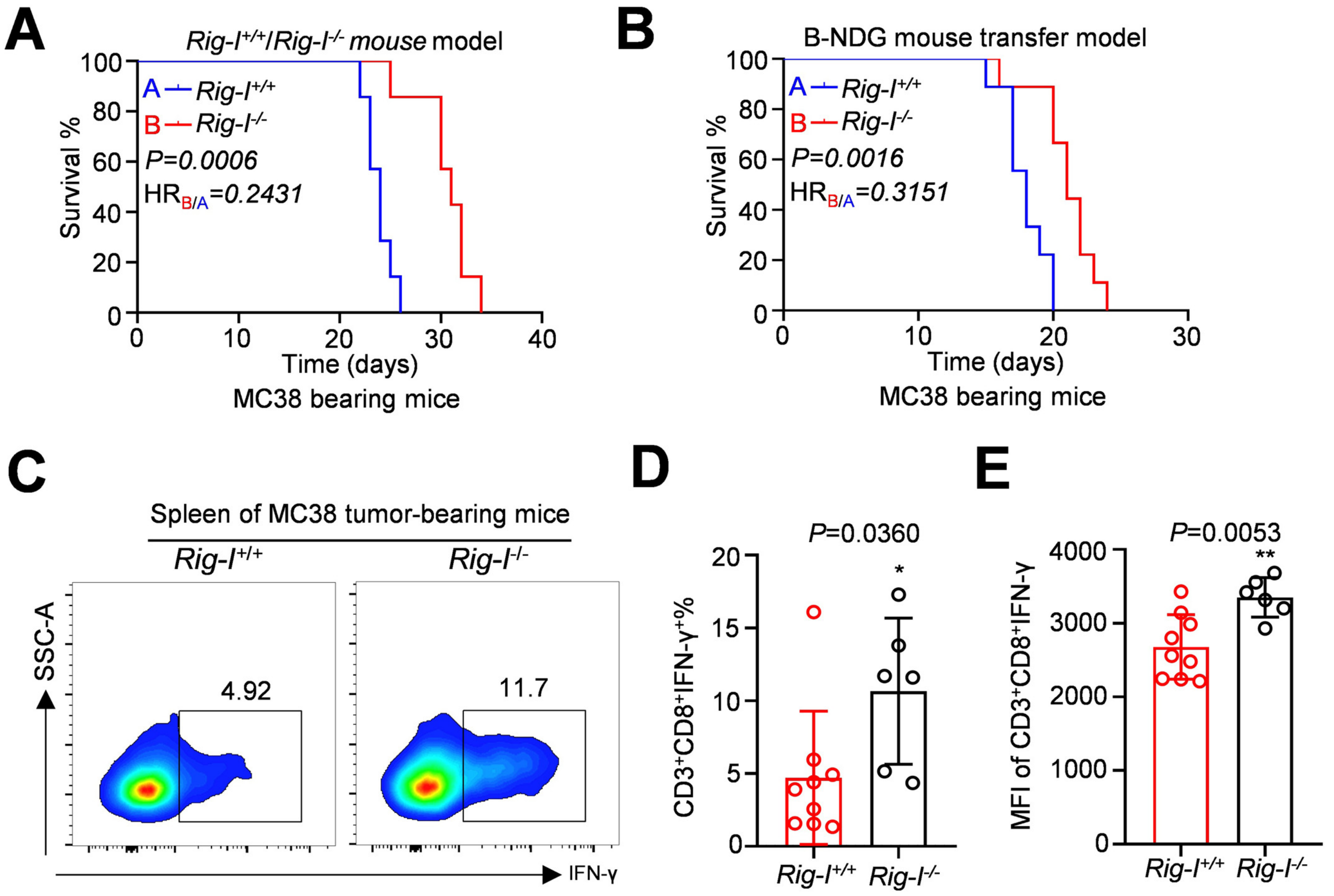

Figure EV3. ***Rig-I* knockout enhanced the anti-tumour function.**

(**A**) Survival curves of MC38 tumour-bearing *Rig-I*$^{+/+}$ and *Rig-I*$^{-/-}$ mice. (**B**) Survival curves of MC38 tumour-bearing B-NDG mice after the adoptive transfer of *Rig-I*$^{+/+}$ or *Rig-I*$^{-/-}$ CD8^{+} T cells. (**C–E**) Flow cytometry was used to detect the proportion (**C**, **D**) and mean fluorescence intensity (**E**) of CD8^{+} T cells that produced IFN-γ in the spleens of tumour-bearing *Rig-I*$^{+/+}$ and *Rig-I*$^{-/-}$ mice after stimulation with PMA/ionomycin and GolgiStop. Data information: The data represent different numbers ($n = 7$ for (**A**) and $n = 9$ for (**B**) of biological replicates. The data represent different numbers ($n = 6$ or 9 for **D** and **E**) of biological replicates and were shown as the means ± SEMs. The log-rank test was used in (**A**) ($P = 0.0006$) and (**B**) ($P = 0.0016$). A two-tailed Mann–Whitney *U*-test was used in (**D**) ($P = 0.0360$). Two-tailed unpaired Student's test was used in (**E**) ($P = 0.0053$). *$P < 0.05$, **$P < 0.01$ and ***$P < 0.001$ compared with the *Rig-I*$^{+/+}$ group. Source data are available online for this figure.

 © The Author(s)

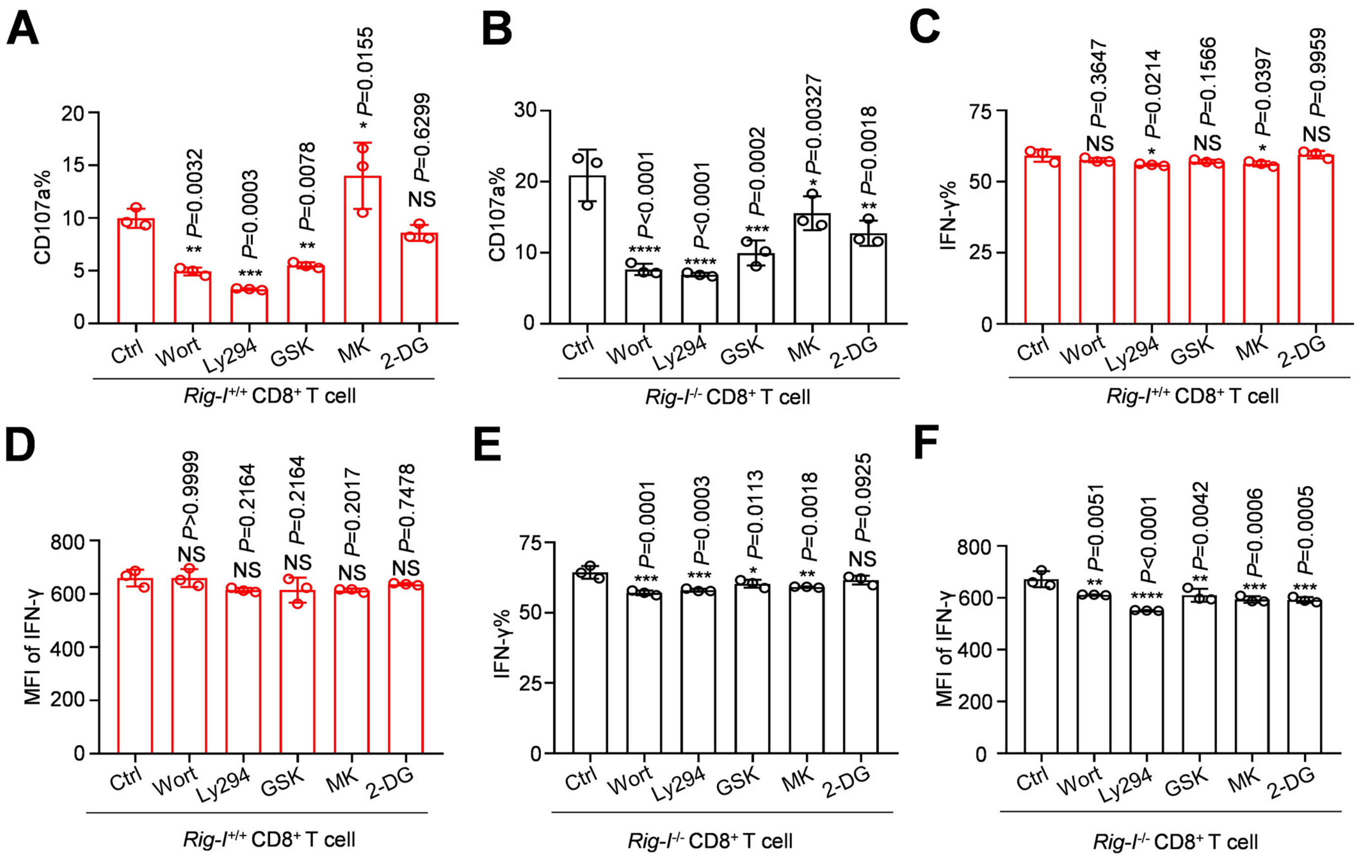

Figure EV4. *Rig-I* inhibited the PI3K/AKT/glycolysis signalling pathway to protect against the anti-tumour effects of CD8⁺ T cells.

(**A–F**). Treatment of *Rig-I*$^{+/+}$ and *Rig-I*$^{-/-}$ CD8^{+} T cells with α-CD3/α-CD28 followed by stimulation with inhibitors of PI3K, AKT and glycolysis. The proportions or mean fluorescence intensities of CD107a (**A**, **B**) and IFN-γ (**C–F**) were detected using flow cytometry after stimulation with PMA/ionomycin and GolgiStop. Data information: The data represented different numbers ($n = 3$) of biological replicates and were shown as the means ± SEMs. One-way ANOVA with Tukey's correction was used for multiple comparisons in (**A–F**) (Exact *p* values were reported on graphs). *$P < 0.05$, **$P < 0.01$, ***$P < 0.001$, ****$P < 0.001$, and NS not significant compared with the Ctrl group. figure. Ctrl control, Wort wortmannin, Ly294 Ly294002, GSK GSK690693, 2-DG 2-deoxy-D-glucose. Source data are available online for this figure.
